# Effect of Different Sand Types and Fractions on Ballistic Resistance of High-Performance Steel Fibre-Reinforced Concrete

**DOI:** 10.3390/ma18215020

**Published:** 2025-11-04

**Authors:** Michal Mára, Jindřich Fornůsek, Tomáš Hrabě, Radoslav Sovják

**Affiliations:** Faculty of Civil Engineering, Czech Technical University in Prague, 166 29 Prague, Czech Republic; maramich@cvut.cz (M.M.); jindrich.fornusek@fsv.cvut.cz (J.F.); tomas.hrabe@fsv.cvut.cz (T.H.)

**Keywords:** ballistic resistance, HPSFRC, sand type, aggregate, fibres

## Abstract

High-Performance Fibre-Reinforced Concrete (HPFRC) is an advanced composite material known for its exceptional energy absorption and dissipation capabilities. To improve its ballistic resistance, HPFRC samples were prepared using 1.5% steel fibre content and varying levels of silica. Ballistic trials employed standard 7.62 × 39 cartridges, each comprising a full metal casing around a mild steel core. Resulting damage and failure mechanisms were mapped using a 3D photogrammetry system. Six different concrete mixtures were tested, each incorporating aggregate fractions of 0/2 mm along with three types of micro sand, the largest of which measured up to 1.2 mm in grain size. The results suggested that increasing the proportion of 0/2 mm silica sand was relatively minor.

## 1. Introduction

In a world facing rising geopolitical tensions and security threats, the need for structures that can withstand extreme impacts—from bullets to blasts—is more urgent than ever [1]. Researchers and engineers are turning to cutting-edge materials like high-performance steel fibre-reinforced concrete (HPSFRC) to build the next generation of protective systems. Cementitious composites, particularly ultra-high-performance steel fibre-reinforced concrete (UHPSFRC), have emerged as a promising material due to their exceptional compressive strength (>150 MPa) and energy absorption capacity. This high-tech concrete with compressive strength exceeding 150 MPa is strong enough to resist the kind of high-speed forces seen in ballistic impacts and explosions [2,3,4,5]. However, optimizing their structural configuration to balance ballistic resistance, economic feasibility, and weight efficiency remains a critical challenge in materials engineering and structural design. Given its unique combination of strength, toughness, and adaptability, HPSFRC represents a highly suitable and forward-looking solution to address modern protective infrastructure demands.

When a projectile strikes cement-based materials, the damage follows a multi-step process. First comes cratering at the point of impact, followed by tunnelling as the projectile moves through the material, and finally rear spalling, where chunks of material break off the back side. This last phase is especially dangerous because it creates high-speed fragments that can be more lethal than the original projectile [6,7,8].

The depth of penetration is strongly influenced by the target’s compressive strength, aggregate hardness and impact factor, which is partly dependent on empirical values and also depends on the compressive strength of concrete and the speed, diameter, and weight of the projectile [7,9,10,11].

Recent studies emphasize that the tensile post-crack behaviour, critical for preventing rear spalling, is significantly enhanced through fibre reinforcement. A 1.5–2.0% volumetric addition of high-strength steel fibres (f_t_ > 3000 MPa) increases fracture energy by 300–400% compared to plain HPC [12,13]. This improvement is attributed to fibre pull-out mechanisms and crack-bridging effects, which dissipate energy and delay structural collapse [14,15,16]. This aspect is particularly important for suppressing the fragmentation of concrete during ballistic damage. Secondary fragmentation during ballistic impact poses a greater risk than the primary projectile, as the resulting fragments have greater injurious potential and occur in significant quantities. Experimental studies by Peng et al. [12] and Wang et al. [13] have shown that dispersed reinforcement (both steel and polymer fibres) significantly improves the cohesion of the composite, especially in tensile areas. Both types of fibres create a bridging effect: after the projectile impact and the formation of primary cracks, the fibres redistribute the load over a larger area, thereby reducing the number and size of secondary cracks.

The way HPSFRC behaves under ballistic impact also depends on how thick it is. In thin panels (≤50 mm), damage usually follows a two-step process: the bullet forms a crater on the front and ejects a cone of material from the back [9,17,18]. In thicker structures, a third phase—tunnelling through the material—becomes important. This tunnelling is directly linked to the projectile’s energy and the concrete’s resistance to shearing forces [10,11,12]. Models developed by researchers like Forrestal [9,17] show how steel fibres can reduce the size of the damage zone by up to 20%. However, accurately predicting the material’s performance under extreme loading conditions remains a complex task—real-world impacts often behave differently than computer simulations because concrete is such a complex, inconsistent material [18,19]

This study addresses some of these aspects through parametric analysis of six types of UHPFRC mixtures with different grain size distributions, combining experimental ballistics (7.62 × 39 mm FMJ projectiles) with 3D photogrammetric damage quantification. The experimental framework evaluates compressive strength (f_c_), flexural strength (f_cf_), DOP and crater surface area across fibre volumes (1.5%) and matrix compositions.

## 2. Materials and Methods

**Figure 1:**
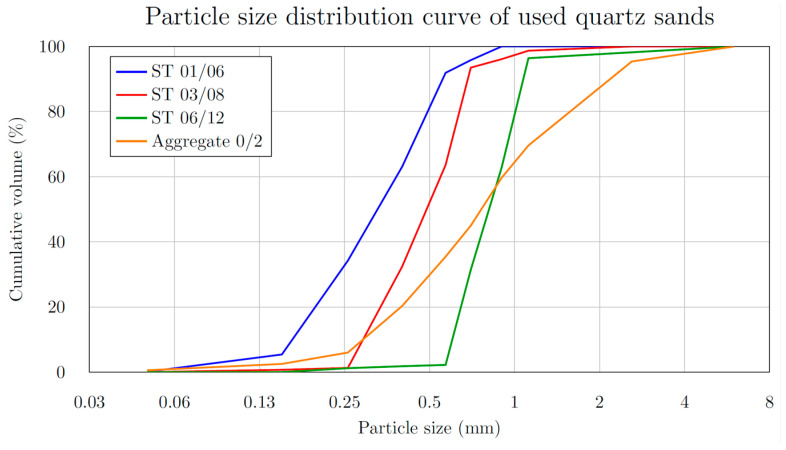
Particle size distribution curve of used quartz microsands and aggregate.

Six UHPFRC mixtures were prepared, each with different grain size curves, aggregate fractions, and sand types. One reference mixture (Mixture A) was used, along with five others (Mixtures B–F) with 0/2 mm technical sand substitutes. For accuracy, granulometric control tests were performed on sands and aggregate fractions, and the results were compared with manufacturer specifications. The graph (Figure 1) presents the particle size distribution curves for the three types of microsands used, as well as for aggregate with grain size fractions 0/2 mm. Granulometric analysis showed that the 0/2 mm sand contained a higher proportion of 0.5 mm grains at the expense of adjacent fractions, but otherwise closely matched the manufacturer’s declared values. From an economic point of view, the price difference between ordinary sand (0/2 mm) and microsand can be up to thirty times, even though ordinary sand still contains a significant proportion of finer grains (Figure 1), which are important for the high compressive strength of the cured composite [20].

**Figure 2:**
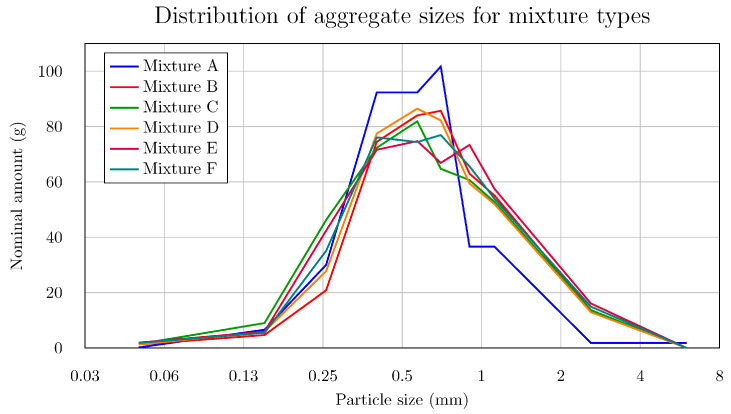
Distribution of aggregate sizes for mixture types.

Table 1 and the graph shown in Figure 2 illustrate the distribution of granulometric content for individual batches as a function of grain size. The data points (Figure 2) are connected to form an approximate envelope representing the proportions present in 1 kg of sand and aggregate. The designation ST01/06 refers to fine construction sand with a particle size range of 0.1–0.6 mm, whereas ST03/08 and ST06/12 denote sands with particle size ranges of 0.3–0.8 mm and 0.6–1.2 mm, respectively.

**Figure 3:**
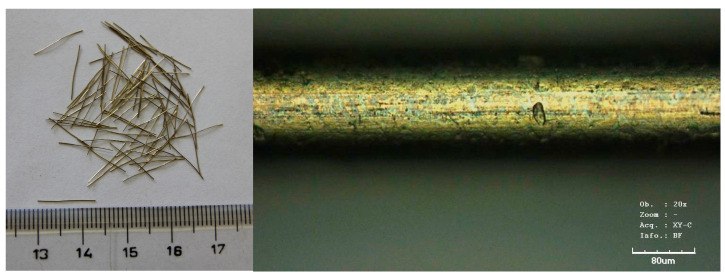
The brass-coated steel fibres.

All mixtures were based on the same overall composition and fine sand content as the reference mixture, with only the aggregate composition and granulometry being varied (Table 2). Individual weight ratios are always expressed as a volume ratio between the given component and cement. The experimental materials consisted of three microsand types (ST 01/06, ST 03/08, ST 06/12) together with an aggregate fraction in the 0–2 mm range, rapid-hardening cement (class 52.5 R), silica fume, quartz powder, superplasticizer, and steel fibres at a volumetric content of 1.5%. Optimized fibre content of 1.5–2.0% by volume was identified as optimal for balancing workability and enhanced performance in the hardened composite [21]. The steel fibres used were straight, brass-coated, and ultra-strong—boasting a tensile strength of 3000 MPa. Their dimensions (0.13 mm in diameter and 14 mm in length) resulted in a high aspect ratio of 108:1, ideal for reinforcing the composite at a microscopic level (Figure 3).

All mixtures were produced using the same procedure. First, the dry ingredients, micro silica, cement, quartz powder, and the various sands and aggregates, were homogenized in a mixer for five minutes. Water and superplasticizer were then added, and the mixture was mixed for another five minutes. Finally, steel fibres were incorporated and homogenized for an additional five minutes. The mixture was formulated as a self-compacting material, enabling it to fill the mould under its own weight without the use of mechanical vibration. Test specimens for ballistic testing measured 300 mm × 400 mm × 50 mm, were demoulded after 24 h, and then cured in water at 20 °C for 27 days. They were then cured in water at 20 °C for 27 days. The mixture was placed in horizontal moulds, which, however, has a negligible effect on ballistic resistance in samples of this thickness compared to placement in a vertical position [22]. The density of the concrete specimens was determined by measuring their mass and volume in accordance with standard laboratory procedures.

### 2.1. Testing

**Figure 4:**
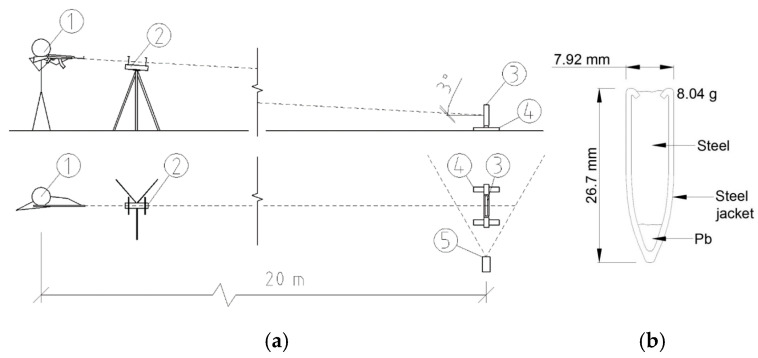
(**a**) Ballistic test setup: (1) Shooter, (2) Optical gates, (3) Test sample, (4) Sample mounting structure, (5) High-speed camera; (**b**) FMJ–MSC projectile, calibre 7.62 × 39 mm.

Each concrete sample was secured in a custom anchoring device. This rig ensured that the sample was tilted exactly 3% off the horizontal axis. To prevent the samples from shifting during testing, clamping screws were used at all four corners, each placed 50 mm from the edges. This setup ensured even pressure distribution and stable positioning throughout the test. The weapon was fired from a distance of 20 m, the standard for controlled ballistics tests. The ballistic test rig also included optical gates for measuring bullet speed and a detailed mounting system to secure the concrete samples (Figure 4a).

The ammunition used was full metal jacket (FMJ) rounds with a steel core—tough and designed to pierce hard surfaces. The bullet itself weighed 8.04 g, with a diameter of 7.62 mm and a total length of 26.7 mm (Figure 4b). The tests were conducted using a CZ 858 semi-automatic rifle—a civilian adaptation of the SA-58.

Measured using optical sensors placed 1 m from the rifle’s muzzle, bullet velocities ranged from 680 to 720 m/s. Using a standard method developed by ballistic expert Kneubuehl [23], the actual impact speed at the concrete surface was estimated to be around 22 m/s lower, accounting for air resistance over the 20 m distance.

The samples were evaluated according to the methodology established by Vossoughi [24] and EN 1523 standards [25]. EN 1523 defines ballistic resistance classes ranging from FB1 to FB7 based on weapon type, projectile weight, impact energy, and velocity. Although this classification was originally intended for security products such as doors and windows, it is now widely accepted for various non-military protective materials.

According to EN 1523, testing includes environmental parameters, distance specifications, projectile type, and even the use of a 0.02 mm thick aluminum foil placed 0.5 m behind the sample to detect any penetrating fragments. If the foil is punctured or visibly damaged, even without complete perforation of the sample, the test is considered a failure.

### 2.2. Scanning of the Damage

**Figure 5:**
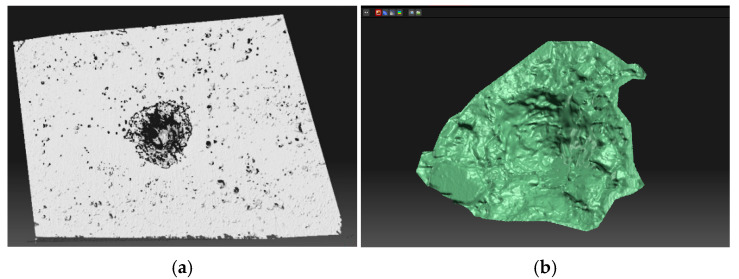
(**a**) SLS-2 David 3D scanner—crater area; (**b**) 3D model of scanned data.

During ballistic testing, specimens were damaged by projectiles, creating truncated cone-shaped craters (Figure 5a). These were scanned from both sides using a 3D scanner based on multi-image photogrammetry. The DAVID SLS-2 scanner (DAVID Vision Systems, Koblenz, Germany), consisting of a high-resolution camera, projector, and processing software (software version 4.5.3, build 1374), enabled capturing up to 2.4 × 10^6^ points per sample. Calibration was performed using a planar glass board, ensuring precision up to 0.1% of the object size. After scanning, data were cleaned, aligned, and merged into a 3D model (Figure 5b), allowing accurate crater surface analysis directly within the software without external post-processing.

## 3. Results and Discussion

**Figure 6:**
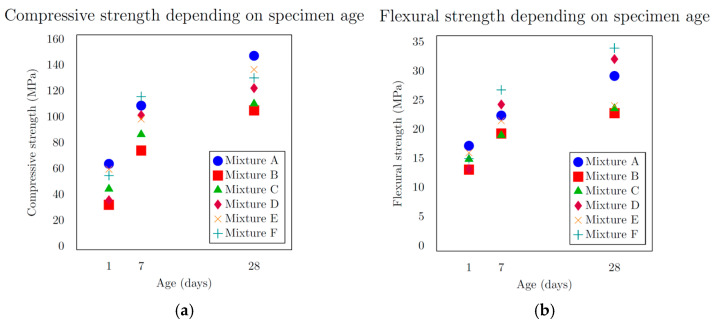
(**a**) Compressive strength depending on sample age; (**b**) flexural strength depending on sample age.

The mechanical test results (Table 3) revealed that mixture A, composed solely of microsands, achieved the highest compressive strength after 28 days, reaching 146.9 MPa. The lowest value, 104.7 MPa, was observed in mixture B, which included 0/2 mm aggregate and microsands with grain sizes from 0.3 to 0.8 mm. Mixture C, which also contained 0/2 mm aggregate and microsands from 0.1 to 0.6 mm, achieved a compressive strength of 109.8 MPa. Mixtures D, E, and F, which incorporated more varied fractions, recorded compressive strengths of 121.9 MPa, 136.3 MPa, and 129.8 MPa, respectively. The results (Figure 6a) indicate, within the scope of this study, that the compressive strength depends on the grain size distribution curve. A better-optimized and more consistent curve, exhibiting a homogeneous and refined particle distribution achieved by limiting the use of a single microsand fraction, resulted in an increase in compressive strength of over 23% The lowest flexural strengths were again found in mixtures B and C (Figure 6b), at 22.7 MPa and 23.5 MPa, respectively. The highest value, 33.9 MPa, was observed in mixture F, while the reference mixture A achieved 29.1 MPa. These results can be attributed to the higher degree of homogeneity and cohesion in the matrix reinforced with steel fibres.

The density of the concrete specimens was determined by measuring their mass and volume in accordance with standard laboratory procedures (Table 4).

**Figure 7:**
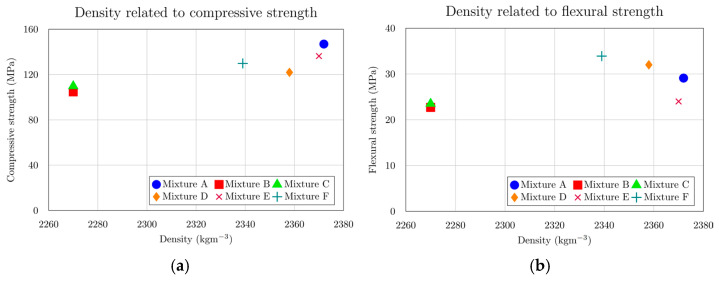
(**a**) Compressive strength at 28th day depending on density; (**b**) flexural strength at 28th day depending on density.

Both charts (Figure 7) examine how concrete density relates to its mechanical properties across the tested mixtures. For compressive strength (Figure 7a), the trend is clear: mixtures with higher density generally reach greater compressive strength values, confirming that a denser microstructure enhances the material’s capacity to resist compressive loads. However, for flexural strength (Figure 7b), the relationship with density is far less pronounced—while there is a slight tendency for higher-density mixtures to exhibit greater flexural strength, the data do not show a strong or consistent positive correlation. This suggests that factors beyond density, such as mix composition or microcracking behaviour, may play a more dominant role in determining flexural performance.

**Figure 8:**
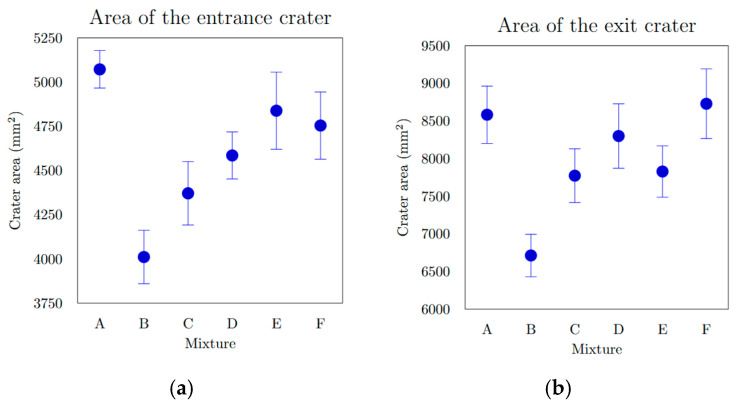
(**a**) Entrance crater area of different mixtures; (**b**) exit crater area of different mixtures.

**Figure 9:**
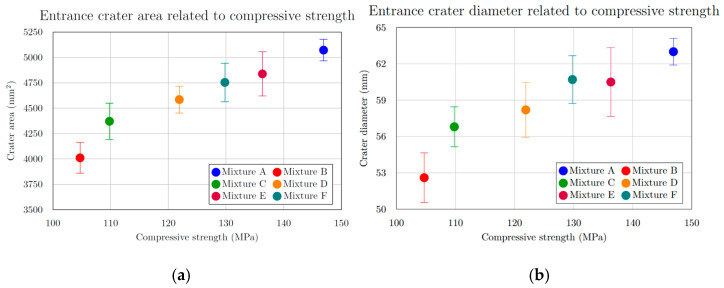
(**a**) Entrance crater area related to compressive strength; (**b**) entrance crater diameter related to compressive strength for mixtures.

**Figure 10:**
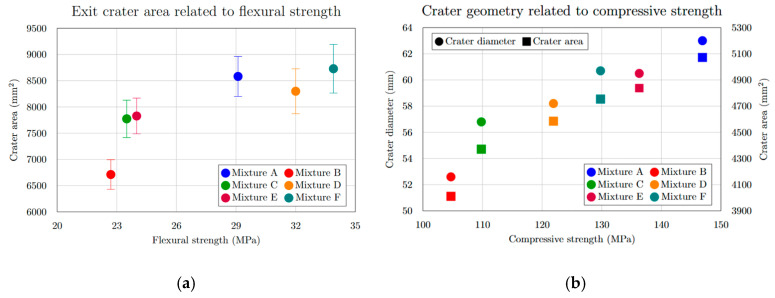
(**a**) Exit crater area related to flexural strength; (**b**) entrance crater diameter and crater area related to compressive strength for mixtures.

The extent of specimen damage (Table 5, Figure 8), determined from the depth of penetration (DOP), showed no notable increase or decreasing trend. All recorded values ranged between 22.6 mm and 25.5 mm, representing less than 6% of the total specimen thickness. A distinct linear relationship was identified between the entry crater diameter and the compressive strength (Figure 9a). With increasing compressive strength, the average diameter of the entry crater expanded from 52.6 mm at 104.7 MPa to 63.0 mm at 146.9 MPa—representing a 16.5% growth in diameter and a 28.7% rise in compressive strength. Similarly, the area of the entry crater (Table 6, Figure 8a) exhibited a proportional increase with compressive strength, varying from 4011 mm^2^ at 104.7 MPa to 5072 mm^2^ at 146.9 MPa. This corresponds to a 21% enlargement of the crater area for a 29% increase in compressive strength. These results demonstrate that compressive strength significantly affects the degree of damage and the composite’s capacity to resist projectile impact (Figure 9b). The enlargement of the crater observed at higher compressive strengths (Figure 10b) can be explained by the material’s enhanced capability to absorb mechanical energy, where a stronger composite absorbs more of the projectile’s kinetic energy, resulting in more concentrated local damage manifested as a larger entry crater. A comparable pattern was found for the exit crater area (Figure 8b) in relation to flexural strength. Up to 25 MPa, the growth in crater area was most noticeable, rising from 6712 mm^2^ at 22.7 MPa to 7828 mm^2^ at 24.0 MPa, an increase of 1116 mm^2^ for only a 1.3 MPa gain in flexural strength (Figure 10a). For the following three mixtures, however, the area increased by merely 900 mm^2^ despite a 9.9 MPa rise in flexural strength. These observations suggest that greater energy absorption in the rear section of the specimen may contribute to the formation of a larger exit crater as the projectile exits the material.

The experimental results demonstrate that replacing part of the microsand content with more readily available and cost-effective 0/2 mm aggregate fractions causes variations in the mechanical performance of the composites, while exerting only a minor influence on their ballistic resistance. In certain mixtures, this substitution even resulted in higher flexural strength and reduced penetration depth.

Overall, the mechanical behaviour of the composites is strongly dependent on the proper gradation and proportioning of aggregate and microsand fractions, with the most favourable outcomes obtained when applying a higher-order polynomial grain-size distribution curve. The best combination of mechanical and ballistic properties was achieved in mixtures incorporating all three microsand types (ST 01/06, ST 03/08, ST 06/12) together with the 0/2 mm aggregate. Conversely, the weakest mechanical performance was observed in mixtures containing only a single microsand fraction (ST 03/08) combined with the 0/2 mm aggregate.

Moreover, the reference composition (Mixture A) indicates that reducing the proportion of sand particles within the 0.9–6 mm range does not significantly deteriorate the composite’s final mechanical characteristics.

## 4. Conclusions

The objective of this study was to examine how various sand types and particle-size fractions affect the ballistic resistance of High-Performance Fibre-Reinforced Concrete (HPFRC). A reference mixture and five other mixtures with different grain size curves were investigated, all containing 0/2 mm aggregate and three fractions of microsand with a maximum grain size of 1.2 mm. The results of the experiments confirmed that replacing part of the microsands with commonly available and more cost-effective 0/2 mm aggregate fractions has only a minimal effect on ballistic resistance. This finding suggests that significant cost savings can be achieved in the production of HPFRC without compromising key performance characteristics.

## Figures and Tables

**Table 1:** Ratios of aggregate and microsands used for mixtures.

Mixture	Aggregate and Microsand Composition [g/kg]			
	ST 01/06	ST 03/08	ST 06/12	Aggregate 0/2 mm
Mixture A	250.0	500.0	250.0	–
Mixture B	–	333.3	–	666.7
Mixture C	333.3	–	–	666.7
Mixture D	100.0	293.0	–	607.0
Mixture E	105.3	175.4	122.8	596.5
Mixture F	103.4	241.4	120.7	534.5

**Table 2:** Composition of reference mixture with defined aggregate ratio.

Composition of the Mixture	kg/m^3^	Weight Ratios
Cement CEM I 52.5 R	695	1
Admixtures	270	0.39
Fine sand	1225	1.76
Water	165	0.24
Superplasticizers (PCE)	40	0.06
Steel fibres	120	0.17

**Table 3:** Mechanical properties of mixtures according to sample age.

Mixture	Compressive Strength [MPa]			Flexural Strength [MPa]		
	1 Day	7 Days	28 Days	1 Day	7 Days	28 Days
Mixture A	63.4	108.4	146.9	17.1	22.3	29.1
Mixture B	31.7	73.7	104.7	13.0	19.2	22.7
Mixture C	44.0	86.1	109.8	14.8	18.9	23.5
Mixture D	35.3	101.0	121.9	13.1	24.2	32.0
Mixture E	59.2	98.0	136.3	15.9	21.4	24.0
Mixture F	54.3	115.4	129.8	14.9	26.7	33.9

**Table 4:** Density of mixtures according to sample age.

Mixture	Density [kg/m^3^]		
	1 Day	7 Days	28 Days
Mixture A	2367	2362	2372
Mixture B	2277	2312	2270
Mixture C	2334	2312	2270
Mixture D	2405	2371	2358
Mixture E	2381	2318	2370
Mixture F	2414	2325	2339

**Table 5:** Depth of penetration and crater diameter of tested samples.

Mixture	DOP [mm]		Diameter of the Front-Side Crater [mm]	
	Average	SD	Average	SD
Mixture A	23.4	0.1	63.0	1.1
Mixture B	23.0	0.8	52.6	2.1
Mixture C	25.5	0.6	56.8	1.7
Mixture D	22.7	0.3	58.2	2.3
Mixture E	24.3	1.2	60.5	2.8
Mixture F	22.6	0.9	60.7	2.0

**Table 6:** Size of the entrance and exit craters for mixtures.

Mixture	Crater Surface Area [mm^2^]			
	Front Side/Entrance		Rear Side/Exit	
	Average	SD	Average	SD
Mixture A	5072	108	8582	381
Mixture B	4011	151	6712	282
Mixture C	4371	179	7773	357
Mixture D	4585	133	8299	428
Mixture E	4838	218	7828	341
Mixture F	4754	190	8728	463

## Data Availability

The original contributions presented in this study are included in the article. Further inquiries can be directed to the corresponding author.

## Abbreviations

The following abbreviations are used in this manuscript:

HPFRC	High-performance fibre-reinforced concrete
UHPFRC	Ultra-high-performance fibre-reinforced concrete
UHPSFRC	Ultra-high-performance steel fibre-reinforced concrete
DOP	Depth of penetration
FMJ	Full metal jacket
MCS	Mild steel core
PCE	polycarboxylate ether
